# Retinal and Cortical Blood Flow Dynamics Following Systemic Blood-Neural Barrier Disruption

**DOI:** 10.3389/fnins.2017.00568

**Published:** 2017-10-12

**Authors:** Flora Hui, Christine T. O. Nguyen, Zheng He, Algis J. Vingrys, Rachel Gurrell, Rebecca L. Fish, Bang V. Bui

**Affiliations:** ^1^Department of Optometry and Vision Sciences, University of Melbourne, Melbourne, VIC, Australia; ^2^Neuroscience and Pain Research Unit, Pfizer, Cambridge, United Kingdom

**Keywords:** blood retinal barrier (BRB), blood brain barrier (BBB), vascular leakage, retinal vessels, cortical vessels, biomarkers, retinal imaging, fluorescein angiography

## Abstract

To consider whether imaging retinal vasculature may be used as a marker for cortical vessels, we compared fluorescein angiography flow dynamics before and after pharmacological disruption of blood-neural barriers. Sodium fluorescein (1%, 200 μl/kg) was intravenously delivered in anesthetized adult Long Evans rats (*n* = 44, brain = 18, retina = 26). In the brain cohort, a cranial window was created to allow direct visualization of surface cortical vessels. Video fluorescein angiography was captured using a rodent retinal camera at 30 frames/second and fluorescence intensity profiles were evaluated for the time to reach 50% brightness (half-rise), 50% decay (half-fall), and the plateau level of remnant fluorescence (offset, %). Cortical vessels fluoresced earlier (artery half-rise: 5.6 ± 0.2 s) and decayed faster (half-fall: 10.3 ± 0.2 s) compared to retinal vasculature. Cortical vessels also had a considerably higher offset, particularly in the capillaries/extravascular space (41.4 ± 2.7%) whereas pigment in the retina reduces such residual fluorescence. In a sub-cohort of animals, sodium deoxycholate (DOC, 0.06 M dissolved in sterile saline, 1 mL) was delivered intravenously to cause simultaneous disruption of the blood-brain and blood-retinal barriers. A separate group received saline as vehicle control. Fluorescein angiography was re-measured at 6 and 24 h after drug infusion and evaluated by comparing flow dynamics to the upper quartile (75%) of the control group. Retinal vasculature was more sensitive to DOC-induced disruption with a higher fluorescence offset at 6 h (47.3 ± 10.6%). A delayed effect was seen in cortical vessels with a higher offset evident only at 24 h (65.6 ± 10.1%). Here we have developed a method to quantitatively compare fluorescein angiography dynamics in the retina and superficial cortical vessels. Our results show that systemic disruption of blood-neural barriers causes vascular leakage in both tissues but earlier in the retina suggesting that pharmacological blood-neural barrier disruption may be detected earlier in the eye than in cortical vasculature.

## Introduction

Given the inaccessibility and the higher cost of imaging the cortical vasculature (Pirko et al., 2005), peripheral sensory organs that have direct connections to the brain are proposed as potential surrogates for the cortex. The retina allows for non-invasive and direct *in vivo* imaging of blood vessels and unmyelinated neurons. Moreover, as the retina shares embryonic origins with the brain (Hoar, 1982; Sinn and Wittbrodt, 2013) with similarities in vascular responses (Strandgaard and Paulson, 1984; He et al., 2012; Tzeng and Ainslie, 2014), it may provide a means to monitor vascular health in the brain (Patton et al., 2005; Frost et al., 2010; Ikram et al., 2012; London et al., 2013).

Changes in the retinal vasculature appears to be a useful indicator of systemic and neurodegenerative diseases (Nguyen et al., 2017). Indeed, retinal vascular abnormalities are manifest in a range of systemic conditions such as diabetes (Hugenschmidt et al., 2014; Wang et al., 2017), hypertension (Wong and McIntosh, 2005; Cheung et al., 2012), and neurodegenerative diseases including multiple sclerosis (Greenberg and Frohman, 2010; Oh et al., 2015) and Alzheimer's disease (Berisha et al., 2007; Cheung et al., 2014; Lim et al., 2016). Furthermore, the presence of vascular lesions in the retina has predictive value for risk of future stroke (Wong et al., 2001; Baker et al., 2008; Cheung et al., 2015, 2017). Conditions known to potentially affect both the brain and retina include hypertension (Fredriksson et al., 1988; Cheung et al., 2012) and multiple sclerosis (Minagar and Alexander, 2003; Correale and Villa, 2007). In addition, hereditary small vessel diseases like cerebroretinal vasculopathy and cerebral autosomal dominant arteriopathy with sub-cortical infarcts and leukoencephalopathy (CADASIL) highlight the developmental similarities between retinal and cortical vasculature where genetic defects lead to significant vasculopathy to retinal and cortical blood vessels only (Kalimo et al., 2002; Hilal et al., 2014; Kolar et al., 2014).

The brain and retina are protected by “barriers,” made up of tight junctions between endothelial cells lining the vasculature. There are many structural and functional parallels between the blood-brain barrier (BBB) and blood-retinal barrier (BRB) (see Nguyen et al., 2017 for review), including the tight junction complexes (Wolburg and Lippoldt, 2002; Goncalves et al., 2013) and carrier-mediated transport proteins (Betz et al., 1983; Tornquist et al., 1990; Steuer et al., 2005; Toda et al., 2011). Thus, the BRB may be a more accessible location to test whether drugs can penetrate the central nervous system (CNS) and thus aid brain-targeted drug discovery. However, the extent to which the BRB functionally mimics the BBB has yet to be directly quantified.

One way to approach this comparison is to quantify the extent of vascular leakage in retinal and cortical blood vessels following pharmacological disruption of the BBB and BRB. Comparisons can be made using fluorescein angiography, a widely used clinical tool for detecting BRB disruption in ocular disease (Ffytche et al., 1980; Patel and Kiss, 2014). For this purpose, we employ a controlled systemic challenge via intravenous delivery of a bile salt, sodium deoxycholate (DOC), which has been previously used to induce barrier disruption in the brain (Spigelman et al., 1983; Zappulla et al., 1985; Greenwood et al., 1991; Seiffert et al., 2004; Tomkins et al., 2007; Prager et al., 2010). To quantify the extent of vascular leakage we quantify dynamic changes in retinal and superficial cortical vessels in rats using video fluorescein angiography (Hui et al., 2014). We test the hypothesis that systemic bile salt delivery will produce similar vascular leakage in both retinal and cortical vessels.

## Materials and methods

All animal care and experimental procedures were carried out in accordance to guidelines from the Association of Research in Vision and Ophthalmology and the National Health and Medical Research Council of Australia (NH MRC, 2004). The protocol was approved by the University of Melbourne Animal Ethics Committee (1111991.1).

### General animal preparation

Adult, male Long Evans rats [18 ± 0.5 weeks, 345 ± 6 g (mean ± SEM), *n* = 44, brain imaging = 18, retinal imaging = 26] were housed in a light (< 50 lux, 12-h light/dark cycle on at 7 a.m.) and temperature (20°C) controlled animal facility with rat chow and water available *ad libitum*.

Surgical anesthesia was induced via inhalation of 3.5% isoflurane (Delvet Pty Ltd, NSW, Australia) and 96.5% oxygen (BOC Ltd, VIC, Australia), and then maintained with 1.5% isoflurane (1.5 L/min). A heated pad (Tempette Junior TE-8J, Techne, New Jersey, USA) was used to maintain body temperature at 37°C. The femoral vein and artery were cannulated using polyethylene tubing (inner diameter 0.28 mm, outer diameter 0.61 mm, Microtube Extrusions, North Rocks, NSW, Australia) to allow intravenous drug delivery and blood pressure monitoring, respectively. As animals were tracked over 2 days, the outlet of the catheters were exteriorized (~2.5 cm left exposed) between the shoulder blades to prevent animal access. Catheters were flushed daily with heparinized saline (200 IU/mL, Sanofi Pharmaceuticals, Macquarie Park, NSW, Australia) to maintain patency.

### Cranial window

As a craniotomy can cause local disruption of the BBB (Easton et al., 1997; Hauss-Wegrzyniak et al., 2002; Xu et al., 2007) we chose to image the vasculature through a thinned skull preparation (Drew et al., 2010; Dorand et al., 2014; Chong et al., 2015). After skin and periosteum removal, the bone was mechanically thinned with a 0.5 mm steel dental bur attached to a rotary drill (Dremel 300S, Robert Bosch (Australia) Pty Ltd, Clayton, VIC, Australia). An area centered ~2 mm anterior to lambda and 2.5 mm lateral from the midline was thinned until the bone became translucent. A constant stream of cooled saline prevented heat damage. A layer of cyanoacrylate glue (Loctite, Henkel Australia Pty Ltd, Kilsyth, VIC, Australia) and dental cement (Rapid Repair self-curing repair material, Dentsply, Mount Waverley, VIC, Australia) was applied leaving only the cortical window visible (Figure S1).

### Fluorescein angiography

Following surgery, animals were transitioned from isoflurane to ketamine and xylazine anesthesia (60 and 5 mg/kg, respectively, Troy Laboratories Pty Ltd, Glendenning, NSW, Australia). All imaging procedures were performed under this injectable combination of anesthesia as it has been shown to have less effect on blood pressure and respiration compared to isoflurane (Albrecht et al., 2014; Tsukamoto et al., 2015). Pupil dilation and topical anesthesia was achieved with tropicamide (0.5%, Mydriacyl, Alcon Laboratories, Macquarie Park, NSW, Australia) and topical proxymetacaine (0.5%, Alcaine, Alcon Laboratories), respectively. GenTeal gel (Novartis Pharmaceuticals, Macquarie Park, NSW, Australia) was used to maintain corneal hydration and acted as a coupling gel for imaging.

**Figure 1:**
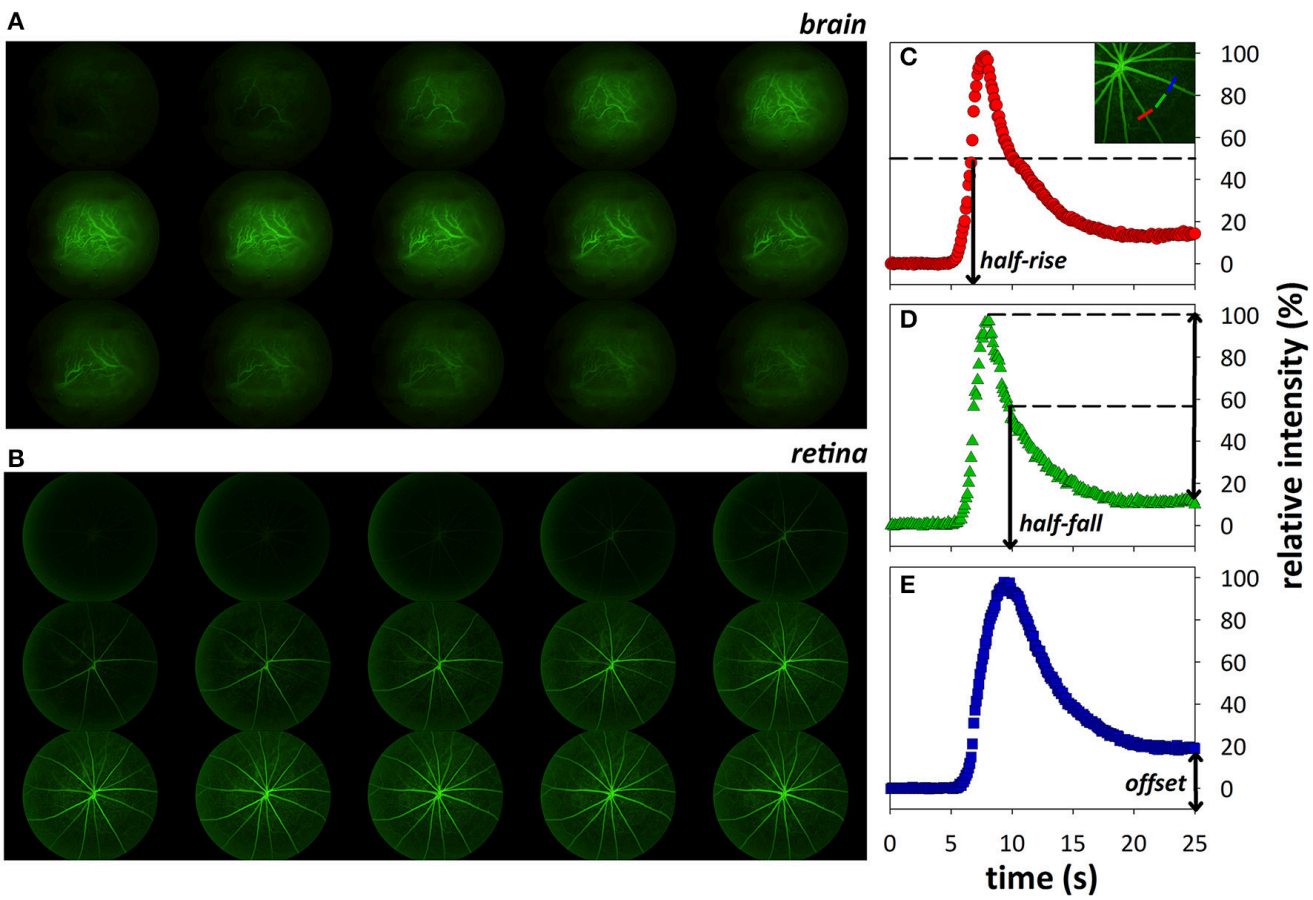
Montage (every 20th frame of video recording) showing fluorescein transit through cortical **(A)** and retinal **(B)** vasculature 5.2–15.8 s after onset of fluorescein infusion. Representative intensity profiles showing how fluorescence indices, half-rise **(C)**, half-fall **(D)**, and offset **(E)**, were derived.

The video fluorescein angiography procedure has been described previously (Hui et al., 2014). Briefly, a bolus of sodium fluorescein (1%, 200 μl/kg, Fluorescite, Alcon Laboratories, NSW, Australia) was infused intravenously (1.05 mL/min over 4 s) via a syringe pump (Harvard Apparatus, Holliston, MA, USA). Video angiography in retina and brain (Figures 1A,B) was recorded at 30 frames/second for 1 min using the Micron III rodent fundus camera (Phoenix Research Labs, USA) and Streampix software (NorPix Inc., Montreal, QC, Canada). Imaged areas were checked for signs of pseudofluorescence prior to injection of sodium fluorescein and no visible signs of pseudofluorescence were observed. Additionally, recordings were initiated 5 s prior to bolus delivery and normalized to this baseline measurement, thus correcting for any potential pseudofluorescence.

### Analysis of fluorescein angiography dynamics

The analysis of fluorescence dynamics used in this manuscript has been described in detail previously (Hui et al., 2014). Briefly, custom MATLAB scripts were written for image registration and analysis of three main parameters: half-rise (time to 50% fluorescence), half-fall (time to 50% decay), and offset (remnant fluorescence expressed relative to peak intensity, %, an indicator of fluorescein leakage; Figures 1C–E).

### Systemic barrier disruption

It is well-documented that topical application of DOC induces local BBB breakdown (Seiffert et al., 2004; Tomkins et al., 2007; Prager et al., 2010). Here DOC was delivered systemically to disrupt both blood-brain and retinal barriers. Given the accessibility of retinal imaging compared to the brain, the eye was used as a platform to examine the dose-response effect of systemic DOC infusion on the vasculature. Three concentrations of DOC were tested (0.01, 0.06, 0.2 M, *n* = 10 per group) to determine the optimal concentration to be used for comparisons between retina and brain. The optimal dose was chosen to be that which produced robust changes in fluorescein dynamics (half-rise and half-fall) and leakage (offset) and was used for comparisons between the retina and brain. In this cohort, animals (*n* = 10 retina, 18 brain) were randomly assigned to receive either 1 mL of DOC or saline (intravenous at 0.033 mL/min for 30 min) under isoflurane anesthesia. Blood pressure was monitored throughout drug infusion. Intravenous DOC infusion impacted blood pressure (see **Table 2**), which could potentially confound fluorescein angiography dynamics. As such, animals were imaged at 6 and 24 h post-infusion, times which are within the 2 days when the effect of DOC is known to manifest (Tomkins et al., 2007).

### Power analysis

Sample size calculation was based on retinal imaging data. Of the parameters used to describe fluorescence dynamics, the offset shows the highest coefficient of variability (retinal capillary offset = 47%). Sample size was calculated based on this conservative value. With a power of 80% for a two-tailed parametric test with an alpha of 0.05, a sample size of 10 would be required to detect a 50% difference between means.

### Statistical analysis

All group data shown are mean ± SEM. Frequency histograms were used to summarize fluorescence dynamics before and after systemic barrier disruption in the brain and retina. Parameters for describing retinal vessel dynamics were bootstrapped (randomly sampled 1,000 times with replacement) to determine the 95% confidence limits. Parameters for cortical vasculature dynamics were compared to these confidence limits to assess for differences between the two locations.

To compare the DOC-induced injury to both the retina and brain, a global index of injury was developed. First, the median and interquartile ranges (IQR, range between lower 25% to upper 75%) were used to quantify the shape and spread of the histograms, respectively. As there have not been similar studies in the past comparing the blood-retinal and blood-brain barriers with pixel-by-pixel image analysis, we sought to develop an index to quantify the amount of injury following DOC administration. As systemic administration of DOC was expected to widely affect blood-neural barriers, the upper quartile (75%) was chosen, to be more indicative of a larger area of injury. For each treated animal, the number of pixels that fell beyond this cut-off was counted and expressed relative to the total number of pixels in the image (%) as a marker to indicate the level of global injury.

## Results

### Cortical vessels fluoresce and decay faster with greater residual fluorescence

**Figure 2:**
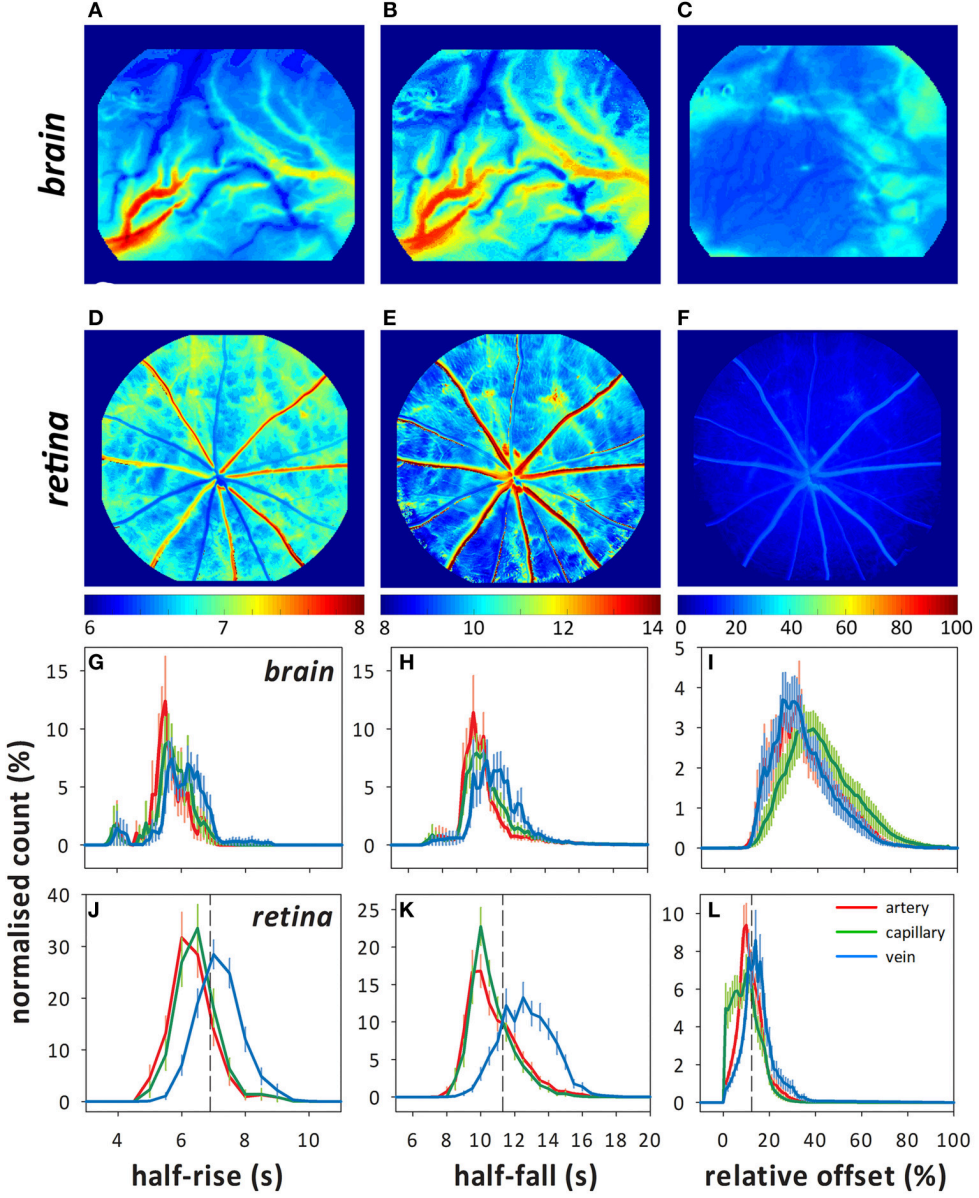
Representative color maps showing the fluorescence indices (half-rise, half-fall, and offset) determined using pixel-by-pixel analysis in the brain **(A–C)** and retina **(D–F)**. Frequency histograms (normalized count to total number of pixels, %) for each index in the brain **(G–I)** and retina **(J–L)** showing the distribution of the fluorescence indices for arteries (red), capillaries (green), and veins (blue). Dashed lines in **(J–K)** indicate where the 75% quartile lies for the capillary data, which is used for calculating the index of injury.

The analysis method identifies arteries (cooler colors) and veins (warmer colors) in both brain and retina by differences in the timing of fluorescein filling (Figure 2, left column) and fluorescence decay (middle column). The offset (right column) appears to be similar between larger arteries and veins in both the retina and brain. This is evident in the overlapping histograms for the arteries and veins (red and blue, respectively, Figures 2I,L), but with increased fluorescence in the capillary/extravascular space in the brain as evidenced by the rightward shift of the histogram in Figure 2I (green).

Compared with the retina, vessels on the surface of the cortex (intraparenchymal vessels) were faster to fluoresce (Figures 2G,J, Table 1, artery median: 5.6 ± 0.2 s, retina 95% confidence limits: 6.4–6.7 s, capillary: 5.8 ± 0.2 s, 6.5–6.9 s, and vein: 6.2 ± 0.2 s, 7.3–7.6 s). Fluorescence decayed faster in cortical arteries (Figures 2H,K, median: 10.3 ± 0.2 s, 10.5–11.2 s) and veins (11.2 ± 0.3 s, 12.7–13.4 s) but not capillaries (10.6 ± 0.2 s, 10.5–11.0 s) compared to the retina. Despite fluorescing earlier and decaying faster, cortical vessels showed a higher offset (Figures 2I,L, artery: 34.7 ± 2.7%, capillary: 41.4 ± 2.7%, vein: 33.6 ± 2.4%) compared to the retina (artery: 11.4–13.8%, capillary: 8.8–11.4%, vein: 14.5–17.2%).

**Table 1:** Distribution of the median and inter-quartile range (IQR) for the half-rise (s), half-fall (s), and offset (%) for each cortical vessel type compared to the bootstrapped 95% CL for the retina.

		**Artery**		**Capillary**		**Vein**	
		**Brain**	**Retina**	**Brain**	**Retina**	**Brain**	**Retina**
Half-rise	Median	5.6 ± 0.2^*^	6.4–6.7	5.8 ± 0.2^*^	6.5–6.9	6.2 ± 0.2^*^	7.3–7.6
	IQR	0.2 ± 0.1	0.2–0.3	0.3 ± 0.1	0.2–0.3	0.4 ± 0.1	0.4–0.5
Half-fall	Median	10.3 ± 0.2^*^	10.5–11.2	10.6 ± 0.2	10.5–11.0	11.2 ± 0.3^*^	12.7–13.4
	IQR	1.2 ± 0.2	1.0–1.4	1.3 ± 0.2^*^	0.8–1.1	1.0 ± 0.1	1.0–1.4
Offset	Median	34.7 ± 2.7^*^	11.4–13.8	41.4 ± 2.7^*^	8.8–11.4	33.6 ± 2.4^*^	14.5–17.2
	IQR	9.3 ± 0.9^*^	4.0–5.7	11.9 ± 0.9^*^	3.9–4.9	10.6 ± 1.1^*^	4.2–6.3

* *Denotes indices that fell out of the retina 95% CL*.

### Retinal fluorescein angiography shows dose-dependent DOC effect

**Figure 3:**
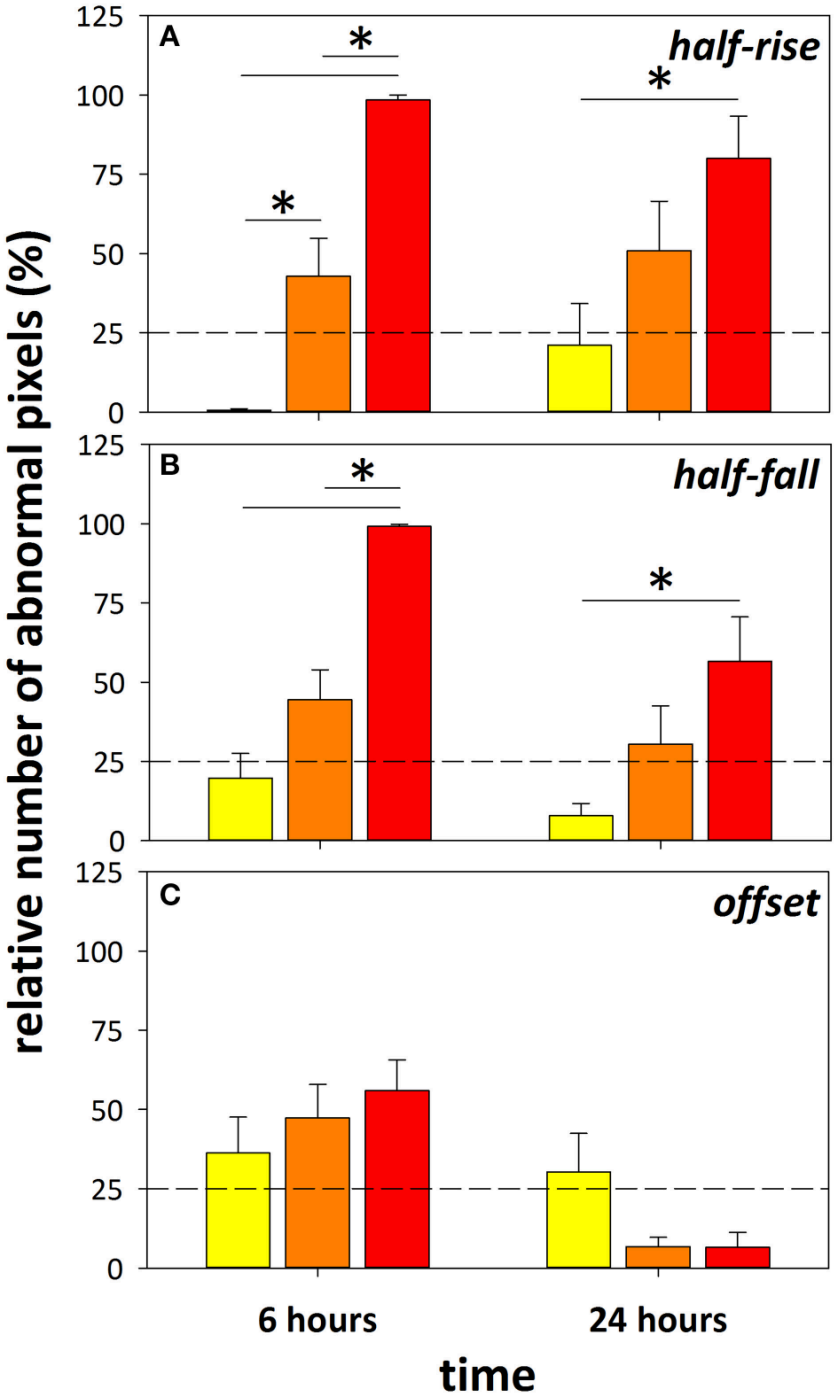
Using the retina as a platform to determine optimal concentration of DOC. Index of injury showing the proportion of pixels (%) that fell beyond a 75% criterion cut-off developed from the control group. Data from eyes treated with 0.02 M (yellow), 0.06 M (orange), and 0.2 M (red) DOC at 6 and 24 h in the half-rise **(A)**, half-fall **(B)**, and offset **(C)** are shown. ^*^Denotes significance between groups with two-way ANOVA and Tukey's multiple comparisons test. Data falling beyond the dashed line marking 25% indicates significant change from control group, mean ± SEM, *n* = 10 eyes/group.

Dose-dependent changes to retinal fluorescein angiography dynamics were evident 6 and 24 h after administration of DOC, as indicated by the index of injury (Figure 3, data for capillaries shown). A similar effect was found in arteries and veins (Figure S2). The lowest dose (0.02 M, yellow) had minimal effect on the BRB. In contrast, at the 6 h time point, 0.06 M (orange) and 0.2 M (red) DOC demonstrated an increased proportion of pixels (or retinal area) with abnormal fluorescein filling, decay, and increased offset (0.06 M: 47.3 ± 10.6%, 0.2 M: 56.0 ± 9.6%). At 24 h, the half-rise and half-fall remained slow but the offset appeared to be significantly dimmer (0.06 M: 6.8 ± 2.9%, 0.2 M: 6.6 ± 4.7%). Although, the 0.2 M dose showed a robust effect, it also induced acute hypertension at 6 h [mean arterial pressure (MAP) = 168.5 ± 1.8 mmHg], followed by hypotension at 24 h (67.6 ± 4.4 mmHg). In comparison, 0.06 M showed more modest effects to blood pressure (6 h: 124.5 ± 4.4 mmHg, 24 h: 81.7 ± 5.6 mmHg, Table 2). Because of the reduced effect on blood pressure whilst still inducing BRB compromise, the 0.06 M concentration was used for comparisons between the brain and retina.

**Table 2:** Average mean arterial pressure (mmHg) in saline control and DOC groups, at baseline, 6 and 24 h after drug administration in the brain and retina cohorts.

**MAP (mmHg)**	**Brain**		**Retina**	
	**Saline**	**DOC**	**Saline**	**DOC**
Baseline	97.2 ± 5.5	94.8 ± 3.8	99.4 ± 3.9	101.5 ± 3.7
Post-infusion	81.6 ± 3.3	86.0 ± 4.0	90.9 ± 4.4	83.4 ± 4.5
6 h	129.7 ± 10.1	113.3 ± 9.6	96.3 ± 6.3	124.5 ± 4.4
24 h	114.6 ± 13.4	78.2 ± 6.1	88.0 ± 6.8	81.7 ± 5.6

*Data shown is mean ± SEM*.

### Retinal vessels are more sensitive to DOC

**Figure 4:**
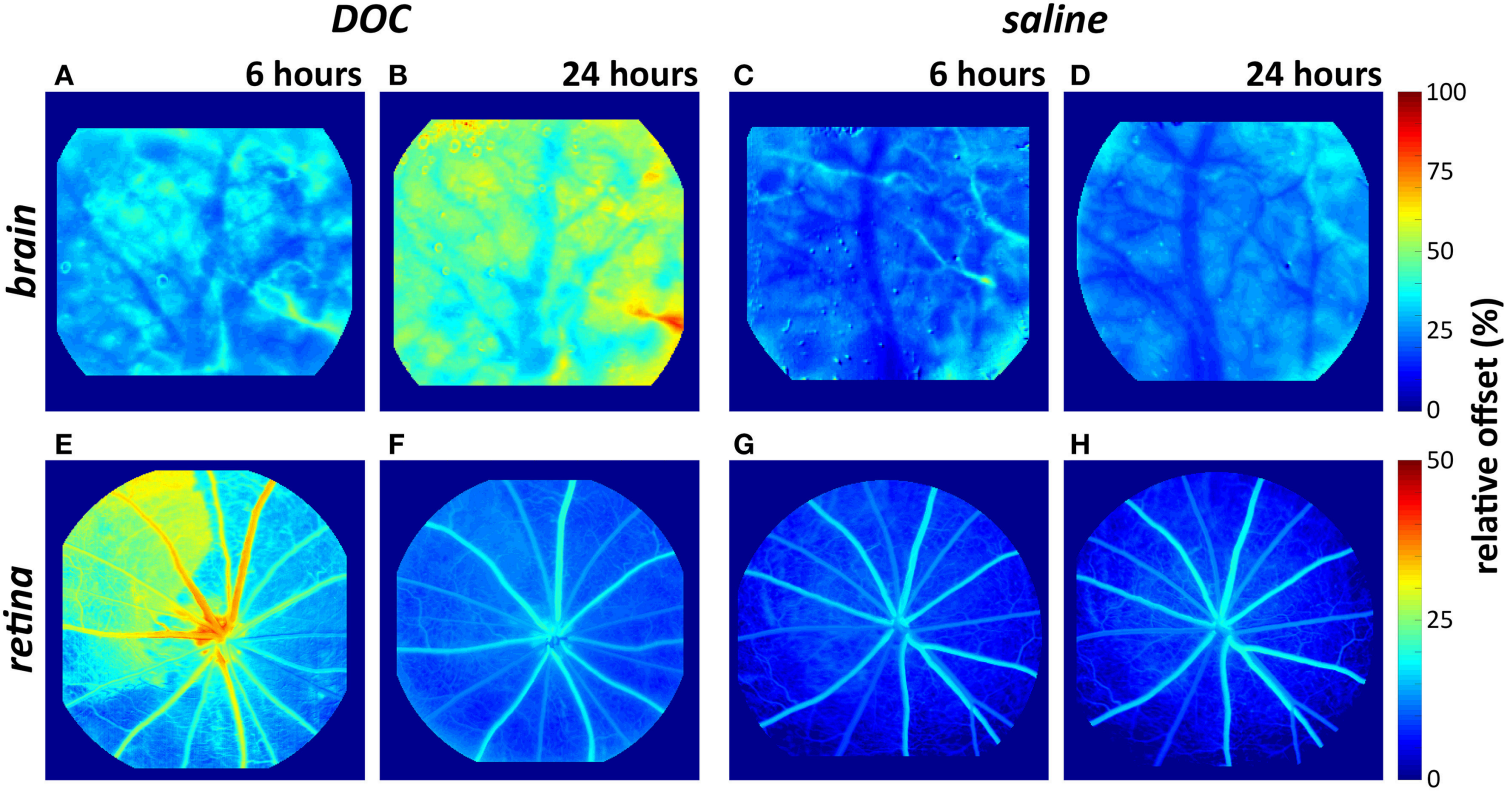
Representative color maps demonstrating changes to the “offset” in the brain **(A–D)** and retina **(E–H)** after DOC or saline infusion. The retinal vasculature showed an elevated offset (i.e., increased fluorescein retention) at 6 h but not 24 h. The cortical vasculature showed an elevated offset 24 h after DOC administration (warm colors in **B**).

**Figure 5:**
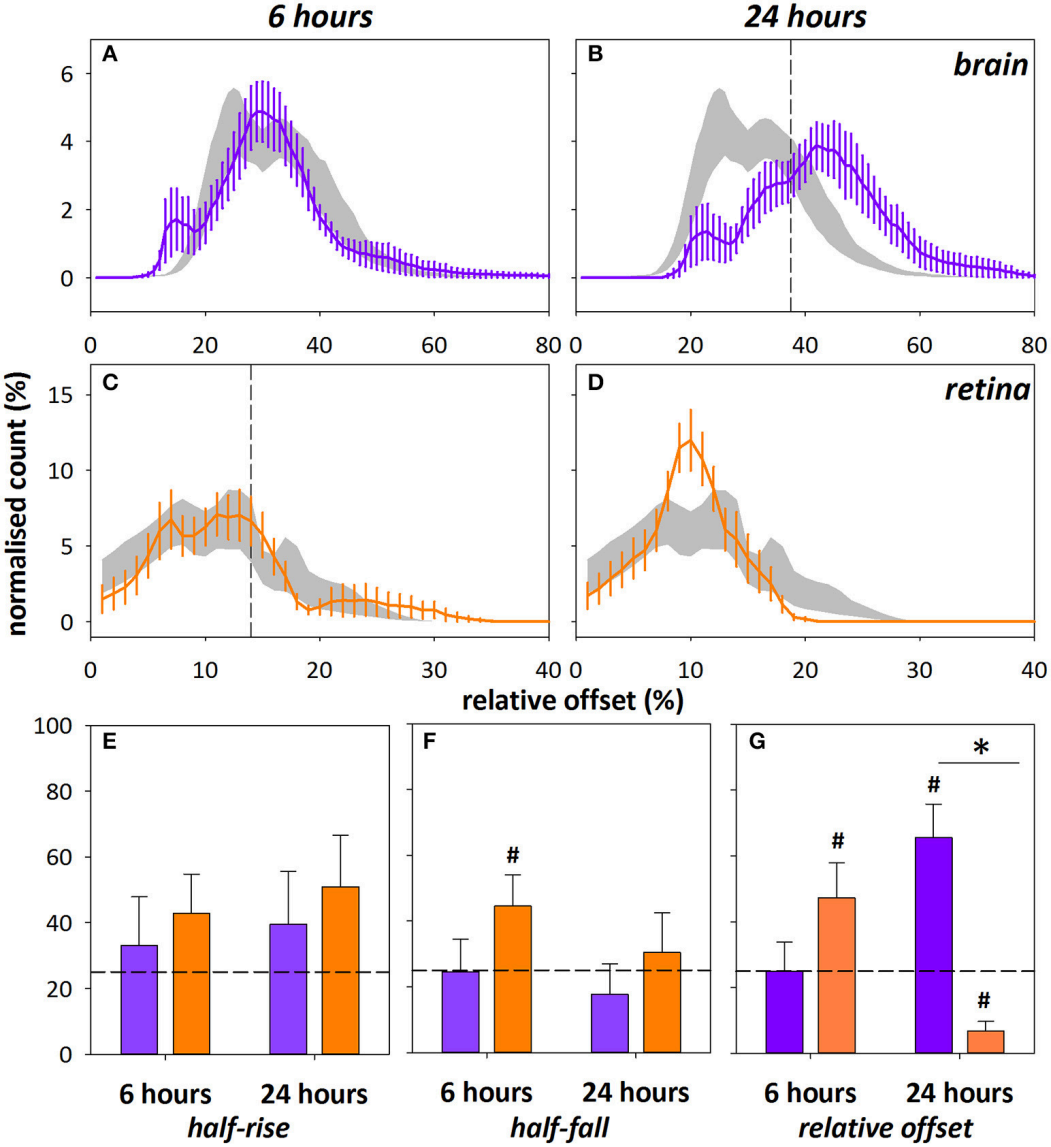
Frequency histograms for relative offset at 6 and 24 h after DOC administration in cortical (**A,B**, purple) and retinal (**C,D**, orange) capillaries/extravascular space. Saline control ± SEM shown in gray, vertical dashed line indicates 75% cut-off determined from the saline control group. Note the different scales for offset between brain and retina. The index of injury (number of pixels above 75% cut-off, %) for the half-rise **(E)**, half-fall **(F)**, and relative offset **(G)** at 6 and 24 h after DOC injury. Data shown for capillaries only, arteries and veins were similarly altered (Figure S3). ^*^Indicates significant difference between groups at 24 h time point, *p* < 0.001, Mann–Whitney test.^#^Denotes indices >±2 SEM from 25% (horizontal dashed line).

Mean arterial pressure did not significantly differ between the brain and eye cohorts at baseline and after 0.06 M DOC infusion [two-way RM ANOVA, *F*_(3, 39)_ = 1.085, *p* = 0.37, Table 2]. Figure 4 shows representative color maps, at 6 and 24 h after infusion of either DOC or saline control. The retinal vasculature was more sensitive to DOC demonstrating increased offset at 6 h (Figure 4E), whereas the cortical vasculature was most affected at 24 h after DOC treatment (Figure 4B). This is statistically confirmed in the frequency histograms of the capillaries/extravascular space (Figures 5A–D), where the retina histograms showed redistribution toward high offset values at 6 h (Figure 5C). On the contrary, offsets higher than controls were not evident in cortical capillaries until 24 h after DOC injection (Figure 5B). Less systematic changes were found for half-rise and half-fall parameters. This is reflected in the index of injury (Figures 5E–G, capillary data shown, other vessel types in Figure S3) highlighting the earlier DOC effect seen in the retina (offset: 47.3 ± 10.6%) compared to the brain (24.9 ± 8.9%, Figure 5G). At 24 h, the converse was true with the brain showing more areas with elevated offset (65.6 ± 10.1%) compared with the retina (6.8 ± 2.9%, Figure 5G).

## Discussion

### Comparing retinal and cortical flow dynamics

We have directly compared *in vivo* cortical intraparenchymal and inner retinal vessels using video fluorescein angiography (Hui et al., 2014) to quantify flow dynamics under normal conditions and in response to blood-neural barrier injury. As retinal and cortical intraparenchymal vessels are at different distances from the heart, with different sized feeder vessels (and thus resistance), it is not surprising that cortical vessels showed faster fluorescein filling (half-rise). The timing of cortical filling however, was similar to previous quantification using contrast-enhanced imaging where Lucifer Yellow dye took on average 5.15 ± 0.15 s to be first detected in pial arterioles and 6.85 ± 0.30 s in pial venules (Prager et al., 2010) compared to 5.6 ± 0.2 and 6.2 ± 0.2 s, respectively in this study. In addition, the cortical surface vessels imaged were significantly larger than the inner retinal arteries (brain: 109.7 ± 13.7 μm, retina: 42.4 ± 3.3 μm) and veins [brain: 155.5 ± 24.6 μm, retina: 50.3 ± 3.4 μm, *n* = 6 per group, two-way ANOVA, *F*_(1, 30)_ = 13.6, *p* < 0.001], as measured using the Diameter plugin in ImageJ (Fischer et al., 2010). Consistent with faster filling, cortical vessels also showed faster fluorescence decay. It is of interest that the difference in half-rise and half-fall for arteries and veins was greater in the retina compared with cortical vessels. Again this would be consistent with a bolus of contrast agent entering and leaving a larger cortical vessel faster than a smaller retinal vessel. This finding may also arise because unlike the retina, cortical vessels visualized through the cranial window do not form a “closed-loop” system (Eberli et al., 1979). Thus, within the small cranial window through which imaging was undertaken, fluorescein filling in a given artery may not drain into any of the visible veins. Furthermore, a cortical vein collects blood from a wider network of venules, many of which may not be visible through the limited window, which can also contribute to faster contrast dye filling.

A higher residual fluorescence (or offset) was also seen in the brain. This is not surprising given the greater depth of cortical tissue and lack of pigmentation, meaning that surface as well as sub-surface vasculature contribute to the measured fluorescence (Prager et al., 2010). Furthermore, the density of capillaries is higher in the cortex (though not homogenously so) compared to the retina (Cavaglia et al., 2001; Arend et al., 2002; McLenachan et al., 2015). The inner retinal vessels have only two capillary beds, in the retinal nerve fiber layer and inner plexiform layer (Paques et al., 2003; Leahy et al., 2015), whilst pigment in the retinal pigment epithelium blocks most of the underlying choroidal fluorescence, which improves the signal to noise ratio when imaging retinal vasculature.

As more retinas were imaged compared to brains, this may have led to less variability in the retinal cohort. It is also possible that the homogeneity of rat retinal vascular anatomy makes it less variable than the brain, consistent with the coefficient of variability (standard deviation/mean × 100) of retinal parameters being lower than in the brain (e.g., vein half-rise: retina = 8.03%, brain = 12.73%). Inherent differences in blood vessel type, diameter, and number between each cranial window preparation is likely responsible for greater variability noted in the brain data.

### Effect of barrier disruption on flow dynamics

The retinal and cortical vasculature were similarly affected by systemic blood-neural barrier disruption, with an elevated relative offset, which is consistent with fluorescein leakage. There was little change in the rate of fluorescein filling (Figure 5E). It is important to note that systemic blood pressure can also affect the offset. Analysis of retinal fluorescein angiography from naïve animals established that higher blood pressure tended to produce a higher offset (offset = 0.13 ^*^ MAP + 1.01, *r*^2^ = 0.37, *p* < 0.001, Figure S4). Given the small increase in blood pressure at 6 h (Table 2) and the slightly lower blood pressure at 24 h, it is unlikely that differences in blood pressure account for the observed elevation of offset and rightward shift of the offset histogram (Figure 5C) post-DOC delivery.

The elevated offset however, is consistent with previous work showing that DOC directly applied to the surface of the brain resulted in increased residual fluorescence with normal filling (Prager et al., 2010). There is a clear indication for a more gradual build-up of DOC related injury in the brain compared to the retina. One explanation for this difference may be because of the heterogeneous vascular beds included in the brain imaging. In particular, as a craniotomy was not performed, the dura and pial vasculature could have contributed to the observed leakage. Notably, two populations of pial vessels have been described: those with similar tight junctions to the cortical vasculature and those with gaps between endothelial cell membranes and thus a more permeable BBB (Allt and Lawrenson, 1997). Pial vessels are also thought to have less astrocyte coverage than intraparenchymal cortical vessels (Bundgaard, 1982; Stewart and Hayakawa, 1994; Cassella et al., 1996; Allt and Lawrenson, 1997; Lawrenson et al., 1999; McCaslin et al., 2011). Given the above, one might expect that a substantial contribution from pial vessels would have the effect of increasing leakage in the brain which occurred at 24 h but not at 6 h post-DOC. We compared the fluorescence profiles of the pial and cortical vessels in our cohort by analysing distinct regions of interest (Figure S5). The pial vasculature could be differentiated from cortical surface vessels as they reside above the cortical vasculature and the camera gave sufficient depth of focus to discern the difference.

Whilst the pial vasculature showed a higher offset at 6 h [two way ANOVA, *F*_(1, 30)_ = 19.79, *p* < 0.001], there was no difference between control and treated groups [*F*_(1, 30)_ = 1.42, *p* = 0.24]. Both pial and cortical vessels showed elevated levels of fluorescein leakage at 24 h after DOC administration compared to control [*F*_(1, 30)_ = 17.89, *p* < 0.001]. In addition, a significant difference was found between vessel types [*F*_(1, 30)_ = 4.42, *p* = 0.04] though no differences were found on Sidak's multiple comparisons test. Given this, it is unlikely that leakage from pial vessels accounts for the earlier leakage in the retina compared with the brain. However, pial leakage may make a later contribution to the higher offset seen later at 24 h.

The earlier leakage of fluorescein in the retina compared with cortical vessels following DOC administration suggests that the BRB is sensitive to systemic disruption of tight junctions. As the imaged retinal blood vessels were generally smaller, preferential earlier leakage is possibly seen in the retina simply due to smaller blood vessels being more susceptible to DOC-induced injury. However, it is also worth noting that we have only assessed leakage at 6 and 24 h, thus how much earlier leakage appears in retinal compared with intraparenchymal vessels remains to be identified. Nevertheless, this shares some parallels with previous studies showing that retinal vascular abnormalities can be indicative of systemic diseases such as diabetes (Wessels et al., 2006; Crosby-Nwaobi et al., 2012; Ding et al., 2012; Wang et al., 2017) and even predictive of other vascular pathology such as stroke (Wong et al., 2001; Baker et al., 2008; Henderson et al., 2011). Here we build on this idea to show that imaging the retinal vasculature may be a sensitive but simpler means to quantify injury to the cortical vasculature.

It is worth considering that the elevated offset may have arisen from damage to the vasculature during surgery to create the cranial window. Whilst the skull thinning process is less invasive than a craniotomy, it can nevertheless cause some trauma to the surface vasculature (Grutzendler et al., 2002; Xu et al., 2007; Yuan et al., 2009; Dorand et al., 2014). Care was taken during surgery to avoid this, including frequent breaks and the provision of a constant stream of cooled saline to minimize friction and heat damage. Also, infusion of DOC/saline only occurred after allowing a day of recovery from surgery. Despite these precautions, some damage may have occurred during the surgical procedure. However, one might have expected that this type of trauma would manifest in relatively increased leakage at the 6 h time point which was not observed (Figure 5G).

Our study has a number of limitations. First, the compromised barriers were probed using fluorescein sodium salt (MW 376.27) leaking through the BRB and BBB. Comparison of a range of fluorescent tracers with different molecular structures and sizes will further define the extent to which the BRB can be used as a surrogate for the BBB. Second, our imaging has been focused on surface cortical vessels, thus care must be taken in extrapolating similarities in retinal vessels to brain vessels other than those superficial on the brain surface. Although, these vessels share many similarities with deeper cerebral vessels in structure and in the way they are affected by bile salts (Greenwood et al., 1991; Seiffert et al., 2004) there are important differences in some of the tight junction proteins (Allt and Lawrenson, 1997) and their associated astrocytes. Another difference has been that two vascular beds have been compared in two separate cohorts of animals. Whilst it would be ideal to image the retina and brain of the same animal simultaneously to allow intra-animal comparisons and remove confounds such as different blood pressures, it was not logistically feasible using our camera system. Fortunately the average blood pressures for the retinal and cortical imaging groups were not significantly different, minimizing possible confounds. Lastly, only male rats were used in this study to remove sex as a potential confound. This is due to inherent differences between male and female physiology, such that they may respond differently to the same pharmacological insult. However, this possibility requires further exploration to ascertain the exact differences that can occur.

Certain eye conditions can lead to BRB compromise without brain and BBB involvement. Indeed, such situations can make it difficult to use BRB changes as a sign of BBB disturbance. Care must be taken to rule out retinal conditions using additional signs and symptoms to avoid confounding this interpretation.

Overall, there are some clear advantages in imaging the retinal vasculature compared to the cortical vessels. First there is no need for any preparatory procedures (i.e., skull thinning or craniotomy). Second, there is lower intra-animal variability for retinal vessels, as there is more homogeneity between the number and diameters of retinal arteries and veins between eyes. Inter-animal variability is also lower as the vasculature is organized in a similar, spoke-like pattern, with two major inner retinal layers, with only minor variations (Figure 1B). In contrast, there is more variability in blood vessel type and size observed in the intraparenchymal vessels (Figure 1A). This is evident in the histograms (Figure 2G) where multiple peaks can be seen in the brain data, likely reflecting the different flow dynamics in differently sized blood vessels. Third, retinal pigment epithelium limits fluorescence from the choroid, thus improving signal-to-noise characteristics for inner retinal vasculature. Finally, the inner retinal vasculature is essentially a “closed loop system,” where blood originating from vessels entering via the optic nerve head will flow into the inner retinal arteries, then to the adjacent capillary bed and drain via neighboring veins that exit via the optic nerve. This allows for robust calculations of circulation times between retinal arteries and veins (Bursell et al., 1992; Arend et al., 2004; Cañas, 2012; Leskova et al., 2013).

In conclusion, we have developed a method to quantitatively compare fluorescein angiography dynamics in the retina and superficial cortical vessels. We show that systemic disruption of tight junctions results in vascular leakage in both tissues. Interestingly retinal leakage was detectable earlier than in the cortical vessels, thus suggesting that the eye may be a sensitive marker for pharmacological barrier disruption of the CNS.

## Author contributions

FH, CN, ZH, BB: Study design, data collection, data analysis, manuscript preparation. AV, RG, RF: Study design, manuscript preparation.

### Conflict of interest statement

CN, BB, AV are joint investigators on an Australian Research Council Linkage grant LP160100126. RG and RF are employees of Pfizer, Neuroscience and Pain Research Unit. The other authors declare that the research was conducted in the absence of any commercial or financial relationships that could be construed as a potential conflict of interest.
