# Supplementary material for: Retinal and Cortical Blood Flow Dynamics Following Systemic Blood-Neural Barrier Disruption
Source: Front Neurosci. 2017 Oct 12;11:568. doi: 10.3389/fnins.2017.00568 (PMC5643486; doi:10.3389/fnins.2017.00568)
Supplement: Supplementary file 1 [file DataSheet1.docx]

**Retinal and cortical blood flow dynamics following systemic blood-neural barrier disruption**

Flora Hui^1^, Christine TO Nguyen^1^, Zheng He^1^, Algis J Vingrys^1^, Rachel Gurrell^2^, Rebecca L Fish^2^, Bang V Bui^1^

^1^ Department of Optometry and Vision Sciences, University of Melbourne, Victoria, Australia

^2^ Neuroscience and Pain Research Unit, Pfizer, Cambridge CB21 6GS, United Kingdom

*** Correspondence:**

Associate Professor Bang Viet Bui

Department of Optometry & Vision Sciences,

University of Melbourne, Parkville 3010

Ph: +61 3 83447006

Email: [bvb@unimelb.edu.au](mailto:christine.nguyen@unimelb.edu.au)

**Supplementary material**


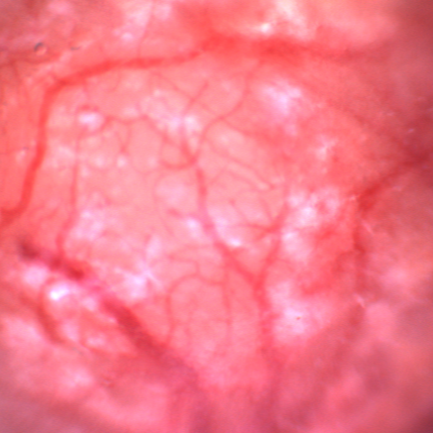


**Figure S1.** Representative image of the brain surface after thinning of the bone to reveal surface cortical vasculature. Scale bar represents 400 μm.

**
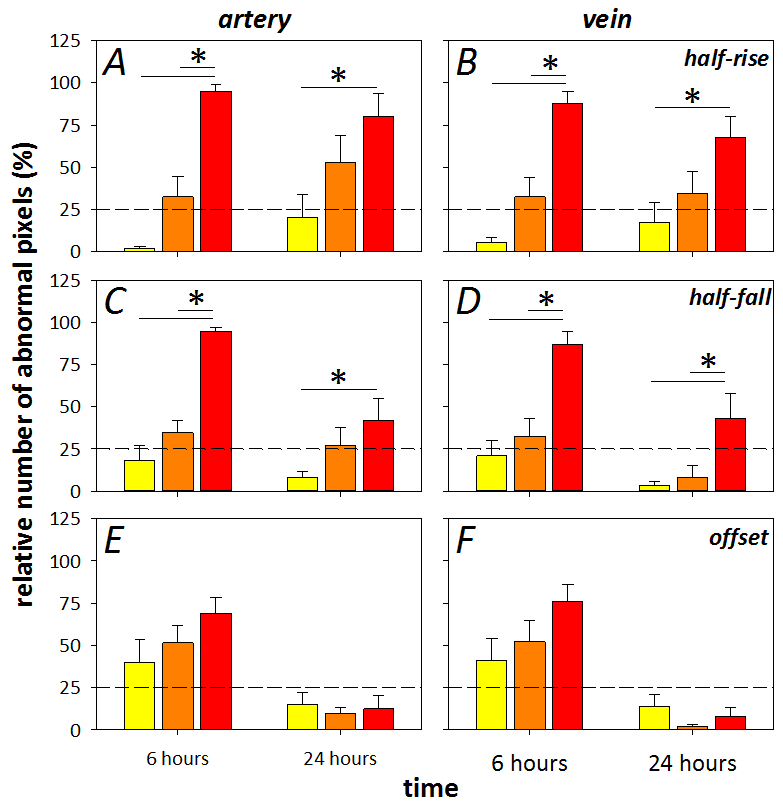
**

**Figure S2.** Index of injury at 6 and 24 hours after administration of 0.02 M (yellow), 0.06 M (orange) and 0.2 M (red) DOC. A 75% criterion cut-off was determined from the control group for half-rise (A-B), half-fall (C-D) and offset (E-F) in arteries (left) and veins (right). * denotes significance between groups with two-way ANOVA and Tukey’s multiple comparisons test. Dashed line shows the 25% mark, data shown is mean ± SEM, n = 10 eyes/group.


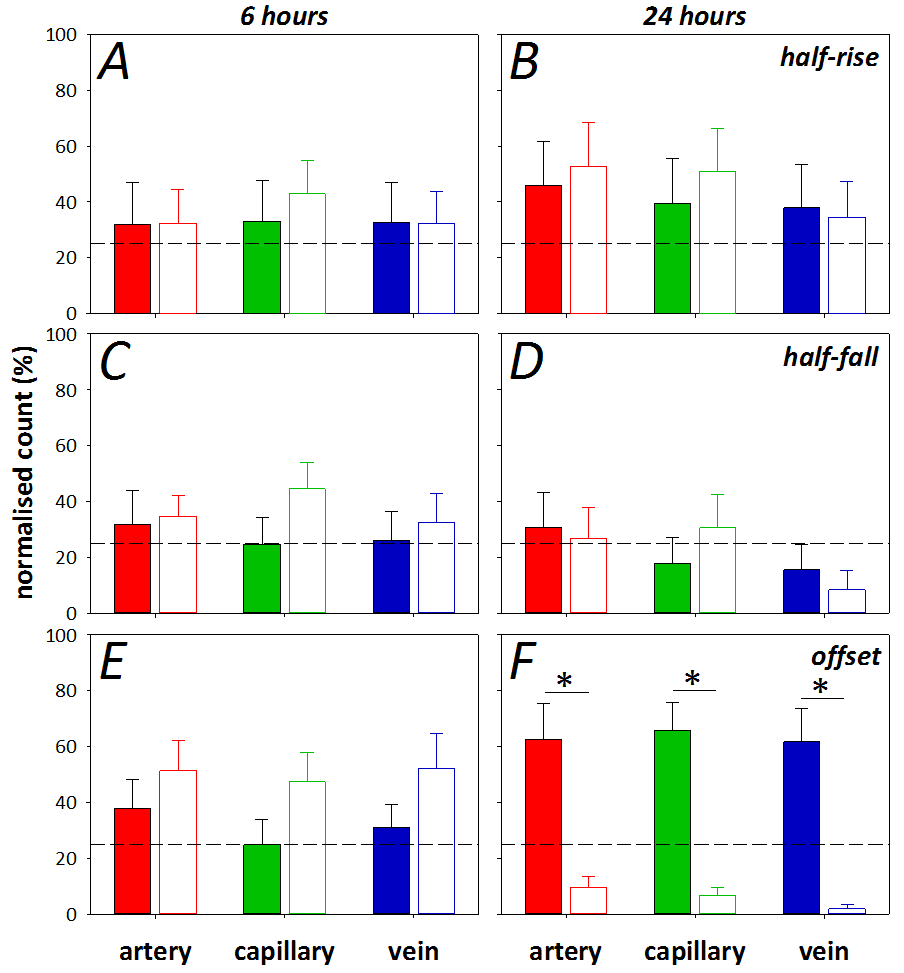


**Figure S3.** Index of injury at 6 (left panels) and 24 hours (right panels) in the brain (filled) and retina (unfilled) after DOC administration, showing the proportion of pixels (%) that fell beyond a 75% criterion cut-off developed from the control cohort. Data shown for the half-rise (A-B), half-fall (C-D) and offset (E-F) and arteries (red), capillaries/extravascular space (green) and veins (blue). * denotes significance between groups using Mann-Whitney Test, p ≤ 0.002.


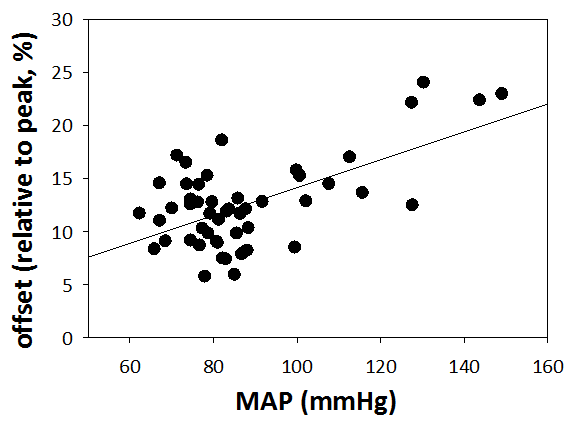


**Figure S4.** Linear regression of the relationship between mean arterial pressure and offset in normal retina (offset = 0.13 * MAP + 1.01, r^2^ = 0.37, p < 0.001, n = 51).


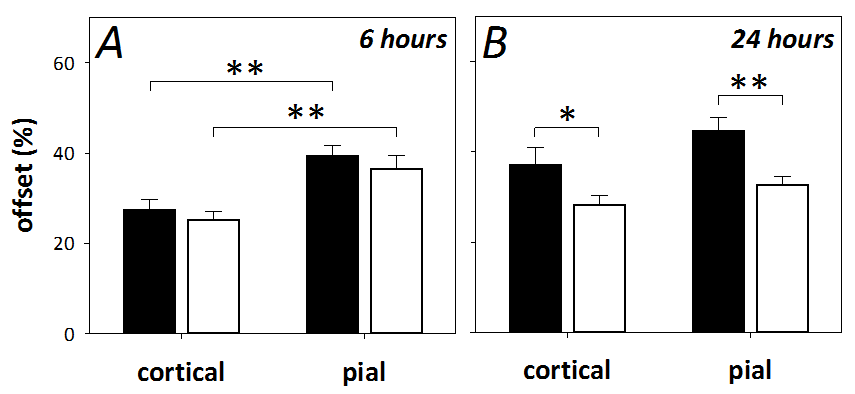


**Figure S5.** Average offset (relative to peak, %) in 4 regions of interest in cortical capillary and pial vasculature, 6 hours (A) and 24 hours (B) after DOC (filled) or saline (unfilled) application. At 6 hours, a significant difference was found between cortical and pial locations but no drug effect is evident. The drug effect manifests at 24 hours; a significant difference was also found between cortical and pial locations, but no difference was found on Sidak’s multiple comparisons test. ** denotes p < 0.01, * denotes p < 0.05.
